# Comprehensive analysis of the immune implication of EPHX4 gene in laryngeal squamous cell carcinoma

**DOI:** 10.1016/j.bjorl.2024.101411

**Published:** 2024-03-07

**Authors:** Nimei Shen, Gang Gao, Xinhong Lu, Jiaxin Jin, Liwei Lin, Maohua Qian, Yang Qin

**Affiliations:** The Second Affiliated Hospital of Nantong University, Jiangsu, China

**Keywords:** Laryngeal cancer, EPHX4, Immunotherapeutic

## Abstract

**Objectives:**

The role of Epoxide Hydrolase-4 (EPHX4), a member of epoxide hydrolase family, has not been investigated in cancer. The purpose of this article is to explore the application value of EPHX4 in laryngeal cancer and its relationship with immune infiltration.

**Methods:**

We observed that EPHX4 expression and its survival assays in laryngeal cancer specimens based on The Cancer Genome Atlas (TCGA) cohorts. We also analyzed the correlation between immune cell infiltration levels and EPHX4 gene copy number in laryngeal cancer. Finally, we conducted in vitro assay to evaluate the functions of EPHX4 in laryngeal cancer cell line.

**Results:**

EPHX4 is highly expressed in laryngeal cancer specimens and has a poor prognosis. EPHX4 related immune cell analysis showed that it participated in NK Natural killer cell mediated cytotoxicity. Finally, Cell experiments indicate that EPHX4 could promote laryngeal cancer cell line proliferation, colony formation and invasion.

**Conclusions:**

Our research results suggest that EPHX4 may be a potential immunotherapy target for laryngeal cancer. The nominated immune signature is a helpful and promising prognostic indicator in laryngeal cancer.

**Levels of Evidence:**

Level 3.

## Highlights

• The expression level of EPHX4 was higher in HNSC patients’ tissues.
• EPHX4 expression is closely associated with the immunity of HNSC.
• EPHX4 promotes HNSC cells proliferation, colony formation and invasion.

## Introduction

Laryngeal cell carcinoma is a complex and malignancy of the head and neck. In the whole world, approximately 150,000 LSCC patients are diagnosed annually.^1^ A recent study showed that more than 11,000 cases of LSCC were diagnosed annually in China.^2^ With the rapid development of surgery, radiation, chemotherapy, systemic therapy and novel gene-targeted therapy, LSCC treatment has improved significantly. Although less than half LSCC patients present with locally advanced disease or regional nodal metastases, the rate of cancer mortality is still high.^3^, ^4^ Therefore, further elucidation of the progression of LSCC at the gene level is urgently needed to improve LSCC patient prognosis.

Accumulating evidences have verified immune infiltrates in the tumor microenvironment of HNSCC.^5^, ^6^ The quantity and function of the immune infiltrate are crucial to induce an efficient antitumor immune response. Various infiltrating immune cells in Tumor Microenvironment (TME) are significantly associated with the different levels of immune response activation, tumor progression and the efficient immunotherapy.^7^, ^8^, ^9^, ^10^ Therefore, knowing the relationship between tumor cells and the immune infiltrate are fundamental for understanding and inventing novel treatments for LSCC patients.

Epoxide Hydrolase-4 (EPHX4) is a member of epoxide hydrolase family.^11^ The function of epoxide hydrolase protein family, which comprises a small number of proteins that act as detoxifying enzymes, mainly catalyzes the addition of a water molecule to an epoxide as the overall reaction in the cell.^11^ Over-activation of epoxy compounds could lead to mutagenic, toxic, and even carcinogenic properties.^12^ EPHX4 is primarily expressed in the brain, however, some studies reported EPHX4 was also expressed in pseudomyxoma peritonei and normal colonic epithelia,^13^ Previous studies have not evaluated the expression level of EPHX4 in LSCC.

In this report, we demonstrated the prognostic impact and the immune implication of EPHX4 in LSCC. To the beginning, we analyzed the relationship between the expression level of EPHX4 and the Overall Survival (OS) of EPHX4 in LSCC patients from The Cancer Genome Atlas (TCGA) datasets. Secondly, we systematically studied the status of lymphocytes and the signaling pathways regulating the EPHX4-regulated immune response. Furthermore, we performed in vitro assay to investigate the function of EPHX4 in LSCC cell lines. Our results showed the potential role of EPHX4 in LSCC would help us to understand the underlying molecular mechanism between EPHX4 and the tumor-immune microenvironment in LSCC.

## Methods

### Data collection and processing

LSCC patients datasets were downloaded from TCGA datasets (https://portal.gdc.cancer.gov/), included 111 tumor and 12 nontumor specimens. The expressing data of mRNAs were analyzed by th the limma package for R software. The R package “survival” was performed to investigate the relationships between LSCC patients and the survival data. All original data are downloaded from TCGA datasets. The experimental study was approved by the Ethics Committee of the Second Affiliated Hospital of Nantong University.

### Determination of tumor-infiltrating immune cells in TCGA laryngeal cancer

We used Cell Type Identification by Estimating Relative Subsets of RNA Transcripts (CIBERSORT) way to qualify and quantify 22 different types of immune cells in tissues (https://cibersort.stanford.edu/). This method mostly depends on a leukocyte gene signature matrix, called LM22.

### Relationships between EPHX4 and tumor immune cell infiltration

Tumor Immune Estimation Resource (TIMER) was applied to understand a comprehensive profile of tumor immune cells of pan-cancer (cistrome.dfci.harvard.edu/TIMER/). We evaluated six immune infiltrates (neutrophils, macrophages, dendritic cells, B-cells, CD4+ T-cells and CD8+ T-cells) in HNSCC by utilizing this website server. Several modules were mainly provided on this website, containing Gene, Survival, Mutation, SCNA, Diff Exp, Correlation, and Estimation. We investigated correlation between immune cell infiltration and survival, EPHX4 copy numbers, EPHX4 mRNA expression levels, and individual immune cell infiltration.

### Immunomodulators

The relationship between immunomodulators and EPHX4 expression were explored from a web portal TISIDB (http://cis.hku.hk/TISIDB/) to explore tumor-immune system interactions. We analyzed immunostimulators and immunoinhibitors that were significantly associated with EPHX4 expression (Spearman correlation test, *p* *<* 0.05). The result of protein network was then subjected to GO annotation and KEGG pathway enrichment analysis utilizing web-based tools (https://string-db.org/) and WEB-based Gene SeT Analysis Toolkit (http://www.webgestalt.org/).

### Cell culture

One human laryngeal cancer cell line-HN6, were obtained from Typical Culture Cell Bank, Chinese Academy of Sciences (Shanghai, China), and cultured in DMEM supplement with 10% FBS (Thermo Fisher Scientific) and 1% penicillin-streptomycin.

### Immunoblotting

Cells were lysed with ice-cold RIPA buffer supplemented containing 125 mM Tris-HCl (Ph 6.8), 4% SDS, 20% glycerol, and 0.004% bromphenol blue supplemented with complete protease cocktail. Protein concentrations were measured with a BCA protein assay kit (Pierce). Proteins in the lysates were separated by SDS-PAGE and immunoblotted.

### RNA isolation and qPCR analysis

Total RNA was isolated with TRIzol reagent (Takara), and cDNA was then produced with a RevertAid First Strand cDNA Synthesis (Roche) and served as template for real-time PCR using an SYBR Green-based qRT-PCR system according to the manufacturer’s protocol. Elative RNA levels of indicated genes were determined using the comparative 2^−^*ΔΔ^CT^* method and normalized to GAPDH.

RT-PCR primers used:

EPHX4 (forward: 5’--3’: TACGTGCGGATCAAGGATTCA, reverse: 5’--3’: GGTAACGCCAAGAATACCAGAA)

GADPH (forward: 5’--3’: GGAGCGAGATCCCTCCAAAAT, reverse: 5’--3’: GGCTGTTGTCATACTTCTCATGG).

### Cell proliferation assay

1 × 10^3^ cells were plated into a new 96-well plate. The cell viability was detected by CCK-8 assay kit (Pierce) following the manufacturer’s protocols at indicated timepoints after seeding. The results were analyzed by GraphPad Prism 8.0 tool.

### Colony formation assay

HN6 or stable cell lines were obtained with 0.25% trypsin, counted, and seeded into a new 6-well dish at 2 × 10^3^ cells/dish. After 10 days, the colonies were calculated after fixed with paraformaldehyde (4%), followed by staining with 1% crystal violet and imaging.

### Wound-healing assay

HN6 or stable cell lines were obtained and seeded into new 6-well plates, The next day, cells were mechanically scratched. We got images at the same wounded region at the indicated timepoints after wounding, and the percentage of wound closure was determined by Image J software.

### IHC

We conducted IHC assays based on the standard protocol previously.^14^ Cancer samples from LSCC patients were sectioned into 4-μm thick slides for the detection into of EPHX4 expression. Staining was conducted following to standard protocol.

An immunoreactive intensity score was calculated by multiplying the staining value with the percentage category value, and finally the average score was calculated from scores of the three independent pathologists.

### Statistical analysis

The proper statistical analyses that were conducted using SPSS and Graph Prime 8.0 software. Unpaired Student t tests was performed to compare between two groups, statistical significance was determined as indicated in the figure legend. *p* *<* 0.05 was considered significant; *p < 0.05; **p < 0.01; ***p < 0.001; Kaplan-Meier plots with log-rank test were used to analyze overall survival data.

## Results

### Upregulation EPHX4 expression and its prognostic value in laryngeal cancer

**Figure 1:**
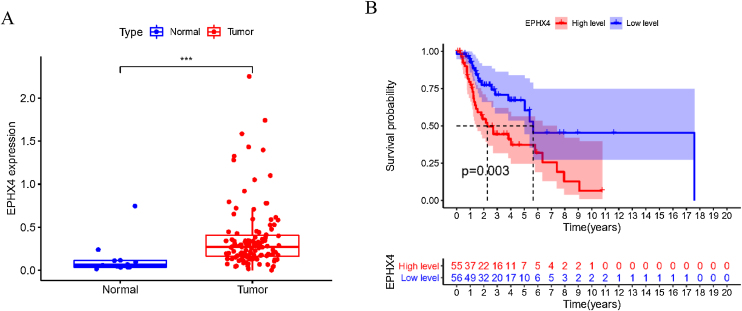
The expression of EPHX4 in laryngeal cancer and its clinical significance. (A) The significant upregulation of EPHX4 was observed in laryngeal cancer. (B) Survival assays based on TCGA datasets. A poor prognosis was observed in laryngeal cancer by TCGA datasets with high EPHX4 expression.

To explore the possible function of EPHX4 in LSCC, we found that the mRNA expression level of EPHX4 was significantly upregulated in LSCC compared with normal tissues (Fig. 1A). Survival analysis based on TCGA datasets revealed that LSCC patients with low EPHX4 displayed a longer overall survival than those with high EPHX4 expression (*p* = 0.003) (Fig. 1B).

### The landscape of infiltrating immune cells in laryngeal cancers and nontumor tissues

**Figure 2:**
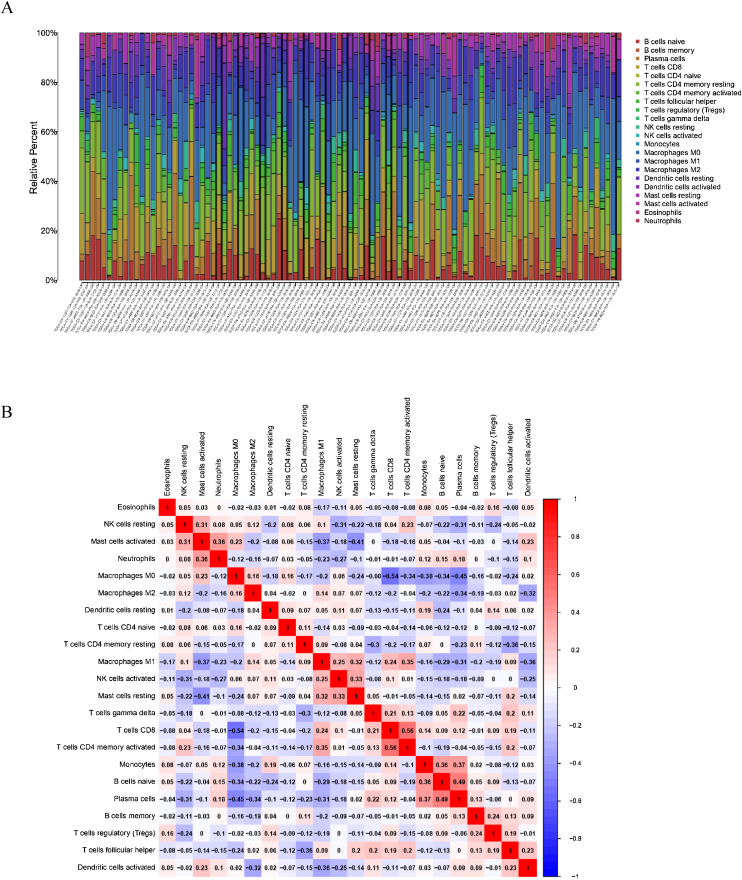
Evaluation of the proportions of 22 types of immune cell infiltration in TCGA cohorts.

**Figure 3:**
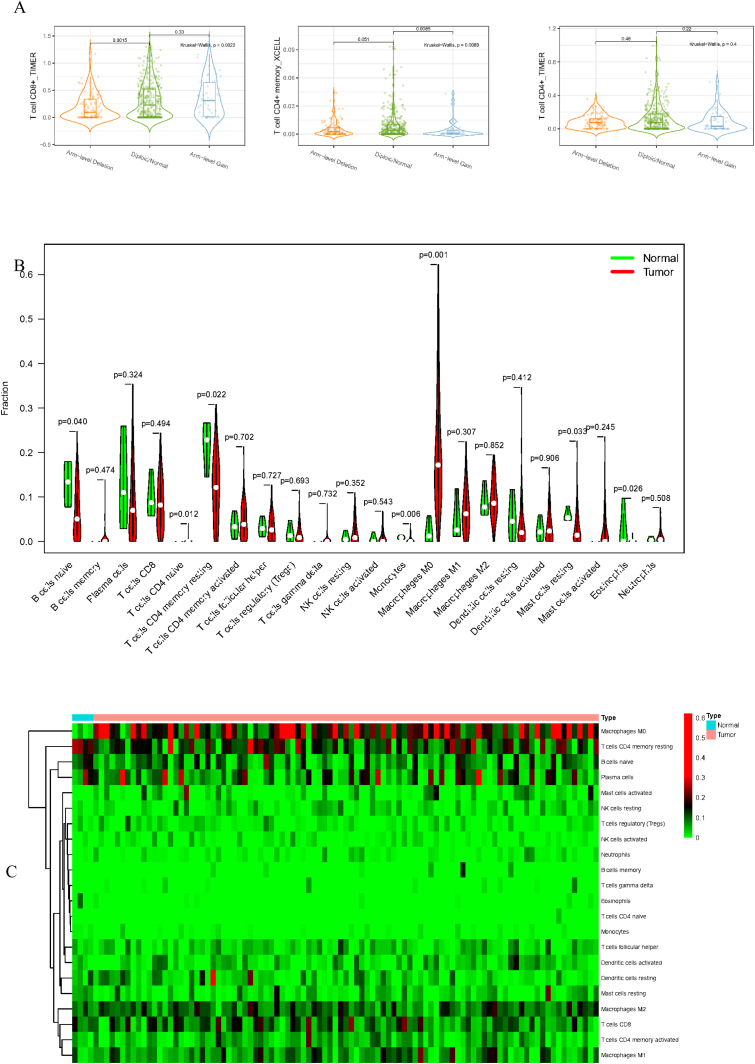
Associations between EPHX4 gene and immune cell infiltration levels. (A) Heatmaps and violin plots showed the differences in the immune cell distribution between malignant (red) and normal (blue) tissue in laryngeal cancer cohorts. (B) The bar chart summarized the percentage of 22 infiltrated immune cells from laryngeal cancer and normal tissues.

To delve into the association of EPHX4 levels with the pattern of immune cells, we extracted and processed the signature gene profile by the use of CIBERSORT method. After removing the samples with *p* ≥ 0.05, the landscape of the infiltrating immune cells in cancerous and nontumor specimens for TCGA LSCC cohorts is showed in Fig. 2. When compared to normal tissues, the proportions of T cells CD4 naive and macrophages M1 were significantly increased, while B-cells naive, T-cells CD4 memory resting, monocytes, mast cells resting and eosinophils in LSCC decreased. In comparison to normal specimens, different patterns of the infiltrating immune cells in TCGA LSCC cohorts are shown in Fig. 3A. Moreover, in TCGA LSCC cohorts, different correlations pattern among the immune cells were found (Fig. 3B). Our results demonstrated that EPHX4 could regulate the immune activity of LSCC microenvironment.

### GO and KEGG pathway

**Figure 4:**
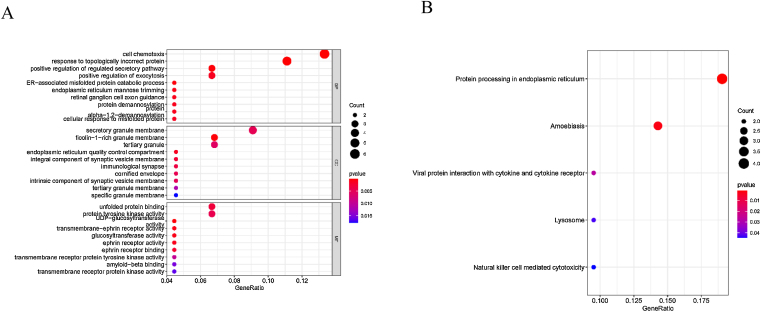
Significant (A) GO and KEGG pathway (B) analysis of the EPHX4 coexpression genes.

We further conducted correlation analysis and found 59 genes significantly related to the expression of EPHX4. Moreover, we conducted GO assays, and Fig. 4A lists the top 10 of each aspect. The enriched BP (Biological Process) was involved in cell chemotaxis, response topologically incorrect protein, positive regulation of regulated secretory pathway, positive regulation of exocytosis, ER-associated misfolded protein catabolic process, endoplasmic reticulum mannose trimming, retinal ganglion cell axon guidance, protein demannosylation protein, alpha-1,2-demannosylation, cellular response to misfolded protein. The enriched CC (Cellular Components) involved secretory granule membrane, ficolin-1-rich granule membrane, tertiary granule, endoplasmic reticulum quality control compartment, immunological synapse, cornified envelope, intrinsic component of synaptic vesicle membrane, tertiary granule membrane, specific granule membrane. The enriched MF (Molecular Function) involved unfolded protein binding, protein tyrosine kinase activity, UDP-glucosyltransferase activity, transmembrane-ephrin receptor activity, glucosyltransferase activity, ephrin receptor activity, ephrin receptor binding, transmembrane receptor protein tyrosine kinase activity, amyloid-beta binding, transmembrane receptor protein kinase activity. We also found five KEGG pathways related to the dysregulation of EPHX4 mRNA expression, which comprised with protein processing in endoplasmic reticulum, amoebiasis, viral protein interaction with cytokine and cytokine receptor, lysosome and natural killer cell mediated cytotoxicity (Fig. 4B).

### Correlations between EPHX4 expression and immune cells

**Figure 5:**
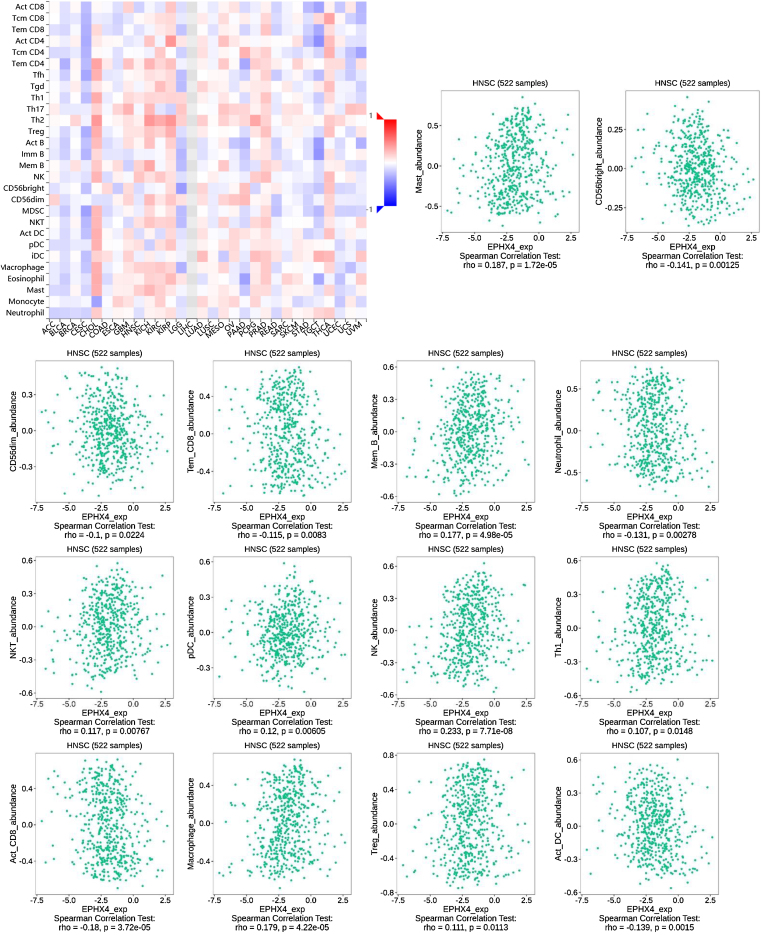
Correlation between EPHX4 expression levels and immune cell subsets. The red and black asterisks in the correlation heatmap indicated various immune cell types significantly associated with EPHX4 expression levels in laryngeal cancer cohorts. The dot plots showed the correlations between EPHX4 expression levels and immune cell subsets in laryngeal cancer cohorts.

Next, we evaluated the correlation between the expression levels of EPHX4 and immune cell infiltration using TISIDB (http://cis.hku.hk/TISIDB/) database, which showed the relationship between EPHX4 expression and abundance of 28 TILs in different types of cancer. Moreover, we found that the expression of EPHX4 is more closely related to the abundance of Mast, CD56bright, CD56dim, Tem_CD8, Mem_B, Neutrophil, NKT, pDC, NK, Th1, Act_CD_8, Macrophage, Treg, Act_DC in HNSCC (Fig. 5).

### Relationship between EPHX4 expression and immunomodulators

**Figure 6:**
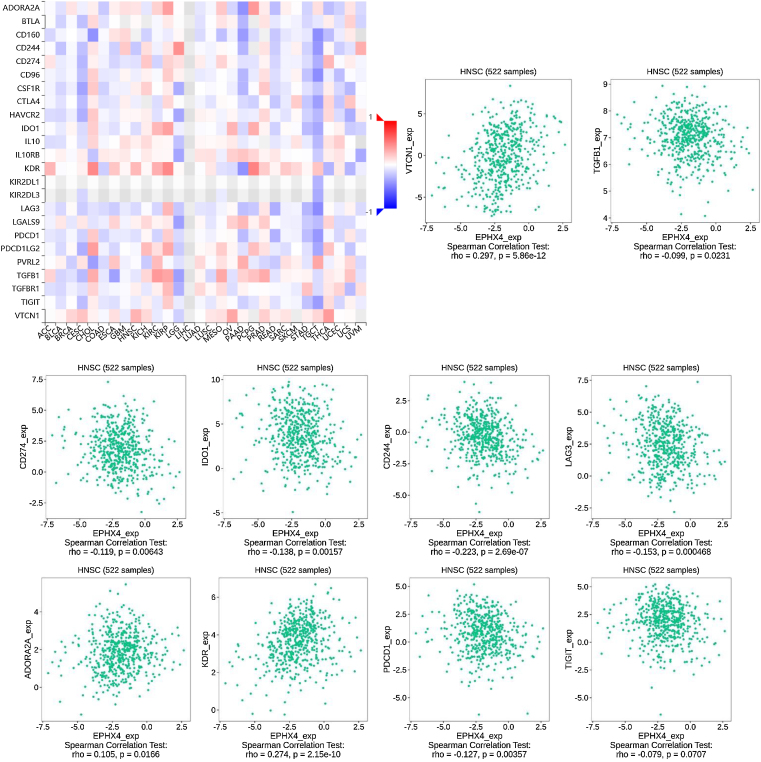
Correlation between EPHX4 expression levels and immunomodulators. The red and black asterisks in the correlation heatmap indicated different immunomodulators significantly associated with EPHX4 expression levels in laryngeal cancer cohorts. The dot plots showed the correlations between EPHX4 expression levels and immunomodulators in laryngeal cancer cohorts.

To further investigate the association EPHX4 expression with immunomodulators, we first studied the relationship between EPHX4 expression and immunoinhibitors among various human cancer types. The results showed that EPHX4 expression was obviously associated with immunoinhibitors in different kinds of cancer. The significant association between EPHX4 and immunoinhibitors in HNSCC was also found. Then, we analyzed the association between EPHX4 expression and immunostimulators and MHC molecule. The results showed that EPHX4 expression correlated obviously with parts of immunostimulators and MHC molecule in HNSCC (Fig. 6).

**Figure 7:**
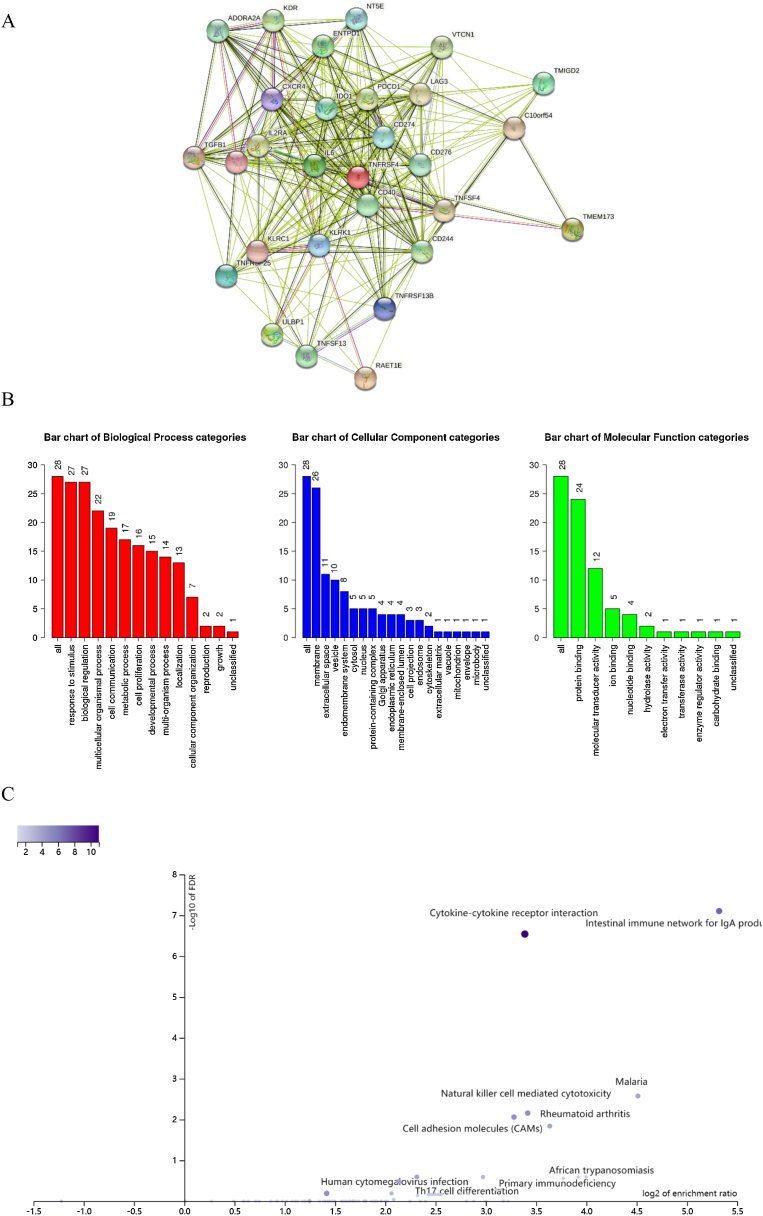
Analysis of immunomodulators associated with EPHX4 expression levels. (A) Protein-protein network of 30-associated immunomodulators in laryngeal cancer, produced by the STRING online server. (B) Gene Ontology annotation of 30-associated immunomodulators in laryngeal cancer. (C) Kyoto Encyclopedia of Genes and Genomes pathway analysis of the above those 30 genes.

We also explored the signaling molecular pathways by which EPHX4 might modulate the immune response in HNSCC. We identified immunostimulators (VTCN1, TGFB1, CDC244, LAG3, PDCD1, TIGIT, IDO1, CD274, ADORA2A, KDR) and immunoinhibitors (TNFSF13, TNFSF4, KLRC1, TNFRSF25, IL2RA, CXCR4, ENTPD1, ULBP1, RAET1E, KLRK1, CD276, TMIGD2, IL6, CXCL12, C10ORF54, NT5E, TNFRSF4, TNFRSF13C, CD40, TMEM173) significantly associated with EPHX4 in HNSCC. we analyzed protein-protein network of 30-associated immunomodulators produced by the STRING online server (Fig. 7A). GO was used to annotate these 30 genes and KEGG pathway enrichment analysis of these gene showed that cytokine-cytokine receptor interaction, intestinal immune network for IgA production, cell adhesion molecules, natural killer cell mediated cytotoxicity, rheumatoid arthritis, Malaria signaling pathway were associated with EPHX4-mediated tumor-immune microenvironment (Fig. 7B,C).

### Depletion of EPHX4 suppressed cell proliferation, clonogenicity, migration in laryngeal cancer

**Figure 8:**
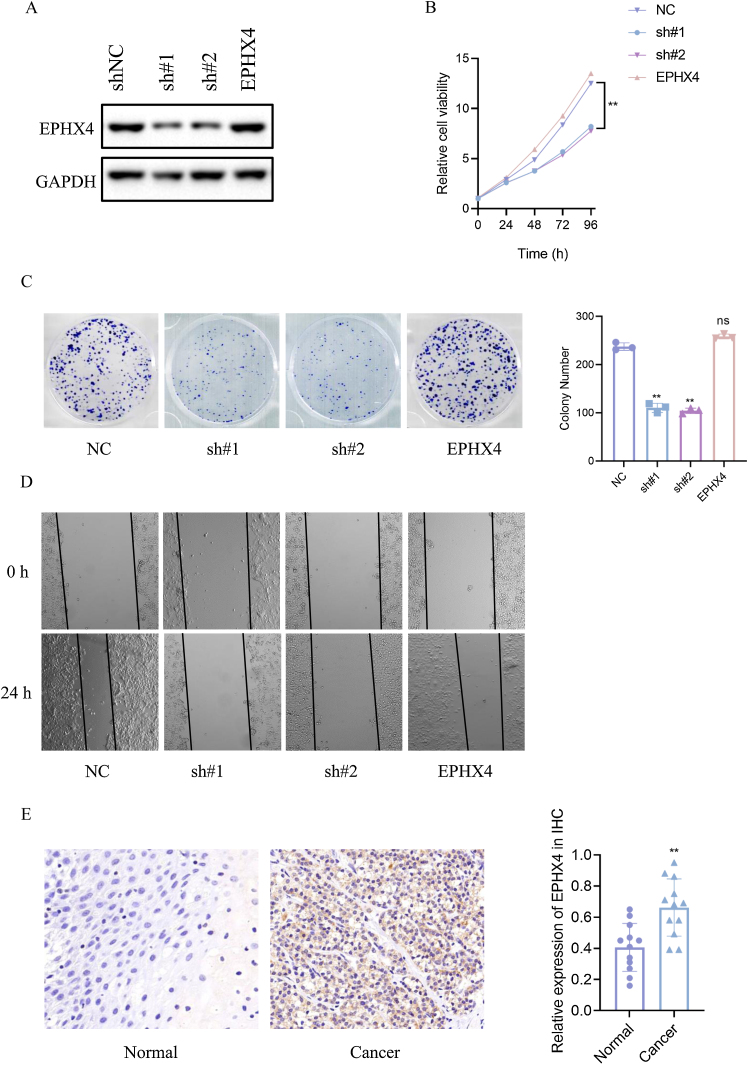
EPHX4 promoted the proliferation, migration of laryngeal cancer cell line. (A) Immunoblotting of normal control (NC) versus EPHX4 knowdown with sh#1, sh#2, and vector versus EPHX4-overexpressing (EPHX4) efficiencies in HN6 cell line. (B) CCK-8 assay was performed in NC and EPHX4 knockdown and overexpression in HN6 cell line. (C) Clonogenic assays of NC and EPHX4 knockdown and overexpression HN6 cell line were recorded (left) and quantitatively analyzed (right). (D) Wound healing assays of EPHX4 knockdown and overexpression in HN6 cell line were recorded. (E) Left: Representative IHC images of EPHX4 protein levels in LSCC tumors and in paired normal tissues.Scale bar:100 uM. Right: EPHX4 protein levels were significantly lower in ESCC tumors than in paired normal tissues (*n* = 12).

To specify the role of EPHX4 gene in LSCC, we then constructed a shRNA-EPHX4 and EPHX4 vector to knockdown and overexpression of EPHX4 respectively in laryngeal cell line (HN6 cell line). Their knockdown and overexpression of efficacy was evaluated by western blotting (Fig. 8A). As determined by CCK8 experiments, EPHX4 knockdown inhibited cell proliferation, while re-expresssion-EPHX4 reversed effects (Fig. 8B). EPHX4 knockdown suppressed colony formation ability, while EPHX4 re-expresssion-EPHX4 also rescued effects (Fig. 8C).

Cancer cell migration plays a key role in cancer progression. To explore the role of EPHX4 in migration, a cell scratch assay was conducted to investigate the horizontal migration ability. Our results showed that migration was inhibited in EPHX4 knockdown cell line, while EPHX4 re-expresssion-EPHX4 rescued. horizontal migration ability of HN6 cell line (Fig. 8D). To explore the expression pattern of EPHX4, we performed an IHC staining to analyze its expression in LSCC tumor tissues, EPHX4 protein was expressed at significantly higher levels in LSCC tumor tissues than in paired normal tissues (Fig. 8E).

## Discussion

LSCC patients in advanced and/or metastatic stagehave a serious poor prognosis.^15^ Recent studies showed that the tumor-immune interactions are critical in the progression of LSCC.^6^ A recent study reported that Orphanin might be a novel oncogenic biomarker for LSCC, which is involved in central and peripheral functions such as modulation of pain and so on. That results suggest the important role of neurons in LSCC progression.^16^ This sobering date suggest the urgent need to find novel biomarkers and explore potential immune-related therapeutic targets for LSCC.

In this research, firstly we evaluate the EPHX4 expression and prognostic of this gene in LSCC patients. The expression level of EPHX4 was higher in LSCC tissues the in normal tissues, and low EPHX4 were significantly associated with better OS in TCGA LSCC cohorts. Altogether, these results demonstrate EPHX4 may act as an oncogenic function and a putative prognostic biomarker for LSCC patients.

Accumulating evidence has showed that there is a great opportunity to predict and guide tumor immunotherapeutic responsiveness due to the constant recognition of the importance of cancer immune microenvironment.^17^ Our study showed that EPHX4 expression is closely associated with the immunity of HNSCC. First, we found that EPHX4 gene copy numbers were significantly associated with the infiltration patterns in HNSCC, such as T-cell CD8+ and T-cell CD4+ memory. Then, TISIDB analysis was performed to investigate the relationship between EPHX4 expression and immune cells, and we discovered that there were significantly positive correlations between EPHX4 expression and abundances of T-cells CD4 naive and macrophages M1, while negative correlations between EPHX4 expression and abundances of B-cells naive, T-cells CD4 memory resting, monocytes, mast cells resting, and eosinophils also were found. Above all results verified that an important role of EPHX4 in cancer immune microenvironment. In this study, high expression of EPHX4 expression predicted a poor prognosis in LSCC. The negative role of EPHX4 expression prognosis of HNSCC patients was consistent to different potential roles of various immune cell types. These findings further suggested that EPHX4 might be an important linkage between progression and prognosis survival of LSCC patients which might act as indicator for LSCC treatments.

Nowadays, increasing evidence verified that immune checkpoint blockade plays a vital role in the treatment for cancer therapy, especially for advanced cancers.^18^ To better understand the underlying molecular and biological function of EPHX4 protein, we performed TISIDB analysis to specify the relationship between EPHX4 and immunomodulator in HNSCC. Most of the immune gene integrated into prognostic signatures involved in the regulation of the activity and proliferation of T-cells, highlighting the importance of T-cell-mediated immunity in HNSCC. However, to our surprise, our results demonstrated that EPHX4 was significantly negatively correlated with CD274, PDCD1, IDO1 and LAG3, which all attenuate anti-tumor immunity and escape destruction by the immune system. Therefore, EPHX4 might be a biomarker for not efficacious therapy based on CD274, PDCD1, IDO1 and LAG3 targets. Our results demonstrated that risk scores based on EPHX4-associated immunomodulators could identify risk groups determined by differential expression patterns of a set of signature genes. Our findings might promote the development of well-defined signatures for LSCC patients’ prognoses.

Using TCGA-HNSCC datasets, a KEGG pathway analysis of EPHX4-associated immunomodulators revealed that natural killer cell mediated cytotoxicity might participate in EPHX4-mediated immune response. The process of T-cell activation needs antigen processing and presentation, NK cell activation is governed by the interaction of NK receptors with target cells. Many positive results are emerging for developing and processing NK cell-based cancer immunotherapy. However, there are many problems to overcome, for example, difficulty to suit clinical-grade ex vivo proliferation, limited in vivo persistence and infiltration to solid tumors, and tumor editing to evade NK cell activity. In our study, it is biologically plausible to speculate that EPHX4 might influence the efficacious of NK-target therapy, further exploring the molecular function of EPHX4 in NK cells is helpful to boost NK-target tumor immunity.

However, this study needs to be expanded to overcome several limitations. Firstly, although we performed in vitro assays to verify the function of EPHX4 in tumor progression, our results were derived from a public database. The clinical prospective research with a larger sample size is urgently required to validate our results. Secondly, we comprehensively studied the immune implications of EPHX4 in HNSCC; however, the molecular mechanism between EPHX4 expression and immune response is still unknown. Further detailed assays should focus on cell and animal research to specify the underlying molecular mechanism of EPHX4 in LSCC samples. Thirdly, we preliminarily explored the relationship between the changes of immunomodulator and EPHX4 expression, a certain number of clinical specimens and co-cultivation assays were still needed to validate our bioinformatics results in the future.

In summary, our results demonstrate that EPHX4 was expressed at high levels in LSCC tissues, and high expression of EPHX4 in LSCC patients predict poorer prognosis. EPHX4 expression was strongly associated with immune characteristics, including infiltrating immune cells and immunomodulators. All these findings, together suggested that EPHX4, might act an important role in the cancer immune microenvironment of LSCC. Therefore, EPHX4 was predicted to be a probable candidate as both a new immunotherapeutic method and a prognostic indicator for LSCC treatment.

## Conclusion

Our study demonstrated that EPHX4 is an oncogene in LSCC patients and compared EPHX4 mRNA expression levels with immune characteristics. We propose that EPHX4 might be a potential immunotherapeutic target for LSCC. In the future study, we will perform in vitro and in vivo experiments to validate the role of EPHX4 in immunotherapeutic value of LSCC patients.

## Funding

The Project supported by Science Foundation of Kangda College of Nanjing Medical University (KD2022KYJJZD021); medical Research Project of Nantong Health Commission (MS2022017).

## Conflicts of interest

The authors declare that they have no known competing financial interests or personal relationships that could have appeared to influence the work reported in this paper.
